# Une polymastie de découverte tardive

**DOI:** 10.11604/pamj.2014.18.193.4900

**Published:** 2014-07-05

**Authors:** Nada El Moussaoui, Mohamed Ait Ourhroui

**Affiliations:** 1 Service de Dermatologie, CHU Ibn Sina, Université Med V, Souissi, Rabat, Maroc

**Keywords:** Polymastie, malformation congénitale, seins surnuméraires, polymastia, congenital malformation, supernumerary breasts

## Image en médecine

La polymastie ou seins surnuméraires est une malformation congénitale mammaire parmi les plus fréquentes. Son incidence est de 0,1%. Elle siège sur une ligne allant du creux axillaire au pli inguinal et traversant le mamelon. La localisation axillaire est la plus fréquente. Il s'agit d'un tissu glandulaire ectopique qui subit les mêmes influences hormonales. Ceci explique la découverte de la tuméfaction par les patientes en période prémenstruelle ou au cours d'une grossesse. Une ablation chirurgicale est préférable vu le risque de dégénérescence. Nous rapportons l'observation d'une patiente de 27 ans, sans antécédent particulier, qui consulte pour une sensation de gonflement au niveau des creux axillaires découverte au cours de l'allaitement. L'examen clinique trouve une tuméfaction axillaire bilatérale de consistance molle, indolore. Une échographie des parties molles réalisée confirmait la nature glandulaire des deux masses. La patiente a préféré l'abstention thérapeutique avec une surveillance régulière

**Figure 1:**
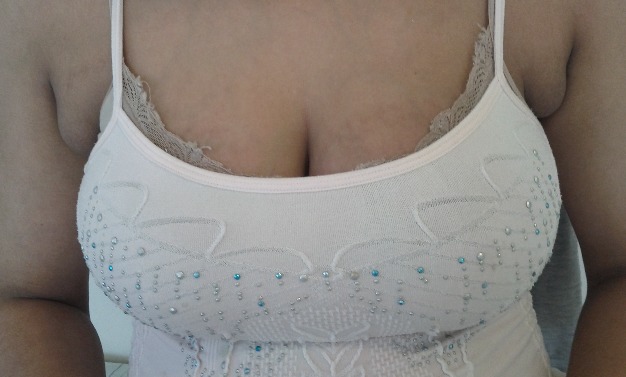
Tuméfaction axillaire bilatérale de consistance molle

